# Supplementary material for: Impact of screen time during COVID-19 on eating habits, physical activity, sleep, and depression symptoms: A cross-sectional study in Indian adolescents
Source: PLoS One. 2022 Mar 8;17(3):e0264951. doi: 10.1371/journal.pone.0264951 (PMC8903250; doi:10.1371/journal.pone.0264951)
Supplement: Attachment — Submitted filename: Response to Reviewer- Ver 2.docx [file pone.0264951.s002.docx]

**Response to Academic Editor and Reviewers**

**Submission ID**: PONE-D-21-24611 R2

**Manuscript Title**: Impact of screen time during COVID 19 on eating habits, physical activity, sleep, and depression symptoms: A cross-sectional study in Indian adolescents

Dear Academic Editor and Reviewer,

Thank you for providing us the opportunity to submit a revised version of our manuscript titled, ‘Impact of screen time during COVID 19 on eating habits, physical activity, sleep, and depression symptoms: A cross-sectional study in Indian adolescents’. We thank reviewer 1 for his constructive feedback and valuable suggestions. We are submitting a point-by-point response to the suggestions provided by the reviewer and the manuscript with revisions highlighted in a colored font. An unmarked revised version of our manuscript is also submitted for consideration at the PLOS ONE journal.

We look forward to hearing from you.

Sincerely,

Authors

**RESPONSE TO REVIEWER 1**

**Reviewer #1: Review round 2 – Screen time

Overall comments:**
Overall, I am very pleased with the effort of the authors during the revisions, and I believe the manuscript has increased tremendously compared to the previous version. I am happy to read that the authors have successfully addressed all my comments. However, there are a few minor points that have to be addressed prior to this study being ready for publication.

***Response:*** *Thank you so much for appreciating our efforts and providing us the opportunity to further strengthen the paper. We are sincerely grateful for your time, expertise, and insightful suggestions that continue to help us tremendously to improve the manuscript. Each of the minor points highlighted by you has been addressed and a point-by-point response to your suggested revisions is provided*.

**General points of interest**
1) I think the authors should carefully check the manuscript for their punctuation. To name some examples: “COVID 19” should be “COVID-19” (Abstract, line 26, line 48; Introduction, line 52…), “some countries, including India had” should be “some countries, including India, had” and there should be a comma prior to each “such as” (for instance line 60 in the introduction).

**Response:** *Thanks for the suggestion. We have made the necessary punctuation-related revisions throughout the manuscript.*

2) the authors should copy-edit the full manuscript for some minor grammatical errors or missing words (for instance “the transmission of coronavirus” should be “the transmission of the coronavirus” (Introduction, line 52-53)

**Response:** *We have edited the text to minimize grammatical errors and improve readability***.**

3) I would suggest to place all reference at the end of a sentence instead of after the part of the sentence the reference relates to. This will improve readability, and the reader can still find the appropriate reference if needed.

**Response:** *Thanks for your suggestion. All in-text citations have been placed in parentheses at the end of the sentences except for a few citations that had to be placed mid-sentence as they reflected the results of multiple studies as a part of a single sentence. For instance, reference 4 was placed mid-sentence, immediately after the finding of the study followed by reference 10 that indicated the findings of another study (Lines 69-70).*

4) Abstract:
I’m glad that the authors have re-written the abstract in a structured way. I think it suits the research better.
I would place the characteristics of the included adolescents in the results section of the abstract (“n=1298, Mage 13.2 (1.1), 53.3% boys”).

**Response:** *As suggested, the participant characteristics have been added in the results section of the abstract (Line 39).*

5) Introduction:
I am happy to see that the authors improved their introduction quite a lot. I however still think the authors could be much more concise as the current introduction still counts 935 words. I would like to challenge the authors to limit the word count of the introduction to at most 600 words (preferably less than 500). The introduction should only focus on letting the reader understand why you did this research, i.e., why is this research important. Also, I would re-arrange the last paragraph of the introduction. I would start with the last sentence (“To the best of our knowledge.”, then the aims and research question (personally, I would not state the research question that specific, but embed it in a normal sentence, such as “We sought to evaluate what the impact of screen time during the COVID-19 pandemic would be on eating habits…”, and thereafter state your specific objectives.

**Response:** *Thanks for your valuable feedback. As suggested, the introduction has been shortened from 934 to ~ 650 words to present a concise summary of the scope, context, and significance of the study. The last paragraph of the introduction has been rearranged and the research questions have been restated as the primary aim of the study (Lines 90-97).*

6) Methods:
I would rephrase the sentence “due to the ongoing … collect data” (lines 118-120) to “An online survey was conducted to collect data, as an in-person survey was not feasible due to the onoing ..”.

**Response:** *Revised.*

7) When explaining the questionnaire concerning screen time. I would suggest omitting the description of specific items, and add the questionnaire to the supplement and refer to that supplement. Now you spend quite a lot words describing the questionnaire, when I will be more concise and also more comprehensive when just referring to the supplement with the questionnaire.

**Response:** *As suggested, we have referred to the questionnaire as supplemental material (line 131) and have provided a summary of the questions, response options, and scoring methods of screen time related items in the main text between lines 130-138.*

8) I think the references after the statement “The PAQ-C/A have been extensively used to evaluate general physical activity levels of children and adolescents in previous studies” (Line 177-179) are missing.
**Response:** *The reference has been added (Line 157, References 24-25).*

9) Results:
“Of 1512 adolescents who provided ….”; I would rephrase this sentence, as the adolescents didn’t provide parental consent, but their parents did. I would suggest “Of 1512 adolescents, of whom parental consent was provided, “

**Response:** *Noted and revised.*

10) In the first paragraph, sometime you use the exact number of adolescents (for instance for the age categories), and sometimes you use percentages (for instance about their living arrangements). I would suggest use the same for the whole paragraph, and I would suggest giving the percentage.

**Response:** *Revised.*

11) Lines 243-244: respectively should always be placed in the end of the sentence (…for study or entertainment, respectively).

**Response:** *Revised****.***

12) For the headings “Comparison of variables between sex and age categories” and “Association of ST on lifestyle behaviors”: omit the first sentence, as this is information you’ll typically find in the methods and distract from the actual results. Also, I would omit “of variables” as this does not add anything to the subheading. Also, try not to explain how certain scores were calculated in the results section, this is also information that should be in the methods and not be repeated in the results.

**Response:** *Noted and revised.*

13) In the methods section you write in the statistical analysis that coefficients are presented as standard coefficient and standard error (SE); I however only see the standardized coefficients in the text, so please add the SE’s as standardized coefficients themselves doesn’t tell us much.

**Response:** *Thanks for your feedback. The standardized regression coefficients and standard error values as mentioned in Table 4 have been added in the text and highlighted in red font (Lines 293-301)*

14) Discussion:
The first paragraph nicely summarizes the main findings of the study. I would however not repeat the exact results (such as the mean ST of percentages), and also not explain here how for instance vigorous PA levels were estimated, as this is already explained in the methods. Try just to repeat the main findings in words rather than in numbers, as you already did that in the results. (For instance, the first key finding could be rephrased as: “First, we found that adolescents use screens for a considerable amount of time, and used their screens more during the week than during the weekend. Second, moderate to vigorous PA levels were reached by only a few adolescents, and was the lowest in girls.”).

**Response:** *As suggested, we have rephrased the key findings to include only the summary of the main results in the first paragraph of the discussion section (Lines 304-309).*

15) Try not to use the word significantly too often. Differences are statistically significant, otherwise you wouldn’t call it a difference, the same applies to an association; if an association would not be statistically significant, you wouldn’t call it an association.

**Response:** *Thanks. The required revisions have been made throughout the paper.*
